# Exploring the Antioxidant Potential of Blackberry and Raspberry Leaves: Phytochemical Analysis, Scavenging Activity, and In Vitro Polyphenol Bioaccessibility

**DOI:** 10.3390/antiox12122125

**Published:** 2023-12-16

**Authors:** Iulia Varzaru, Alexandra Gabriela Oancea, Petru Alexandru Vlaicu, Mihaela Saracila, Arabela Elena Untea

**Affiliations:** Feed and Food Quality Department, National Research and Development Institute for Biology and Animal Nutrition, Calea Bucuresti, No. 1, 077015 Balotesti, Romania; alexandra.oancea@ibna.ro (A.G.O.); alexandru.vlaicu@ibna.ro (P.A.V.); mihaela.saracila@ibna.ro (M.S.)

**Keywords:** blackberry leaves, raspberry leaves, antioxidants, free radical scavenging, lipid peroxidation, polyphenols bioaccessibility

## Abstract

The goal of this research was nutritional evaluation through the phytochemical analysis of blackberry and raspberry leaves, the screening of their biological activity (antioxidant capacity and inhibition of lipid peroxidation), and the investigation of the effect of in vitro gastrointestinal digestion (GID) of blackberry and raspberry leaves on the bioaccessibility of polyphenol subclasses. The concentrations of the analyzed liposoluble antioxidants were higher (*p* < 0.05) in blackberry leaves compared to raspberry leaves, while a significant (*p* < 0.05) higher content of water-soluble antioxidants was registered in raspberry leaves (with a total polyphenol content of 26.2 mg GAE/g DW of which flavonoids accounted for 10.6 mg/g DW). Blackberry leaves had the highest antioxidant capacity inhibition of the superoxide radicals (O_2_^•−^), while raspberry leaves registered the highest inhibition of hydroxyl radicals (^•^OH), suggesting a high biological potency in scavenging-free radicals under in vitro systems. The maximum inhibition percentage of lipid peroxidation was obtained for blackberry leaves (24.86% compared to 4.37% in raspberry leaves), suggesting its potential to limit oxidative reactions. Simulated in vitro digestion showed that hydroxybenzoic acids registered the highest bioaccessibility index in the intestinal phase of both types of leaves, with gallic acid being one of the most bioaccessible phenolics. The outcomes of this investigation reveal that the most significant release of phenolic compounds from blackberry and raspberry leaves occurs either during or after the gastric phase. Knowledge about the bioaccessibility and stability of polyphenol compounds during digestion can provide significant insights into the bioavailability of these molecules and the possible effectiveness of plant metabolites for human health.

## 1. Introduction

The blackberry (*Rubus fructicosus*) and raspberry (*Rubus idaeus* L.) are plants that belong to the genus *Rubus* spp. of the *Rosaceae* family, mainly distributed throughout the temperate zone of the northern hemisphere [1]. The blackberry plant is native to North America, while the cultivation of raspberry plants originated in Europe [2]. Blackberry and raspberry can easily adapt to different environmental conditions such as climate and soil, but their chemical composition may vary with a series of factors that include cultivation, region, weather conditions, ripeness, the time of harvest, and storage conditions [3,4]. 

Berries are the main commercial product of the blackberry and raspberry and are an abundant source of bioactive compounds, such as vitamins (C, A, E, B1, B2, B3, B6, and K), organic acids (citric, malic), phenolic acids (derivatives of cinnamic and benzoic acids), polyphenols, minerals, sugars, and dietary fiber [5,6]. Due to the presence of these nutrients, berries have essential positive effects on the human diet and health, such as lowering cardiovascular diseases, improving insulin response, glucose, and lipid metabolism, as well as antioxidant and anti-inflammatory properties [7,8,9,10]. Their phytonutrients can reduce the risk of chronic diseases and improve their management when consumed as part of a well-balanced diet [11]. 

The leaves of berry crops are considered agro-waste or by-products [4,12,13], with an increased content of phenolics and enhanced antioxidant properties compared with berries [14]. Using agri-food waste products, such as leaves, is an important alternative for obtaining raw materials with significant economic potential [15,16]. Moreover, applying the circular economy model to the food production chain can bring financial and environmental benefits [17].

Blackberry and raspberry leaves have long been used in traditional medicine. The leaves of the blackberry are recommended as a relaxant for uterine muscles, for its beneficial effects during pregnancy [18], for their anti-diarrheal effects, and as an astringent for similar enteric disorders, as well as anti-inflammatory properties in infectious diseases of the oral and pharyngeal mucosa [19]. Extracts of raspberry leaves have been used for their anticancer, antioxidant, antimicrobial, and relaxant properties [20], for treating fever, influenza, diabetes, diarrhea, and colic pain [21], and for the relaxation of the uterus during childbirth [22].

Oxidative damage to cellular components such as lipids and cell membranes due to the harmful effects of free radicals is believed to be associated with many degenerative diseases [23]. It has been shown that raspberry and blackberry fruits can act in a synergistic manner to reduce oxidative stress [24]. Blackberry leaves are known to eliminate free radicals that damage cells and are also involved in the protection and strengthening of immunity, as well as lowering the risk of cancer [25]. Raspberry leaf extracts have protective effects on UVB-induced skin photodamage [26].

The aim of this study is the phytochemical analysis of blackberry and raspberry leaves and the screening of their biological activity (antioxidant potential, free radical scavenging, and the inhibition of lipid peroxidation) for their possible utilization as an alternative to synthetic antioxidants and preservatives in the food and feed industry, and also in pharmaceutical and cosmetic industries. Moreover, the studied leaves may be considered an unconventional and inexpensive source of antioxidants for animal nutrition, with potential effects of increasing the antioxidant level in obtained animal products and a positive impact in controlling the lipid oxidative processes that occur in foods during storage time.

## 2. Materials and Methods

### 2.1. Plant Material

The experimental material consisted of leaves from the blackberry (*Rubus fructicosus* L.) and raspberry (*Rubus idaeus* L.) originating from wild-growing plants and collected from 3 different sites located in Olt County (44°26′00″ N, 24°22′00″ E), Romania. Samples were collected in August 2022. 

Each batch of collected material was individually subjected to natural drying at a constant weight and was then transformed via grinding and sieving into a fine powder. Average samples were formed and kept in the dark until the determinations were made.

### 2.2. Analytical Standards

The following phenolic standards were purchased from Sigma-Aldrich (St. Louis, MO, USA): ellagic acid (95%), syringic acid (98%), epicatechin (96%), 4-hydroxy-3-methoxy-cinnamic acid (95%), rutin (95%), vanillic acid (95%), 3-hydroxybenzoic acid (95%), protocatechuic acid (96%), caffeic acid (95%), coumaric acid (98%), epigallocatechin (97%), catechin (95%), quercetin (95%), resveratrol (99%). Ferulic acid (97%) and chlorogenic acid (95%) were purchased from European Pharmacopoeia (EP). FAME (fatty acid methyl esters) as a standard mixture was purchased from Merck (Darmstadt, Germany). The standards for carotenoids and vitamin E isomers were purchased from Sigma-Aldrich (St. Louis, MO, USA): lutein (95%), zeaxanthin (95%), astaxanthin (97%), canthaxanthin (95%), α-tocopherol (96%), γ-tocopherol (96%), δ-tocopherol (95%). 

### 2.3. Proximate Analysis

The proximate composition of the blackberry and raspberry leaves was determined as follows: crude protein (ISO 5983-2/2009) using the Kjeldahl method (Kjeltec auto 1030 Tecator Instruments, Höganäs, Sweden), crude fat (SR ISO 6492/2001) via continuous solvent extraction (Soxtec 2055 Foss Tecator, Höganäs, Sweden), crude fiber via the method with intermediary filtration (Fibertec 2010 System Foss Tecator, Höganäs, Sweden), dry matter (ISO 6496/2001) and ash (ISO 2171/2010) using the gravimetric method and a Nabertherm calcination furnace (Nabertherm GmbH, Lilienthal, Germany).

### 2.4. Minerals Analysis

The content of copper, iron, manganese, zinc, and calcium was determined via flame atomic absorption spectrometry (FAAS) after microwave digestion (Berghof, Eningen, Germany), using Thermo Electron SOLAAR M6 Dual Zeeman Comfort (Cambridge, UK) equipment, as described previously by [27]. Phosphorus was determined using a colorimetric method and a UV–Vis spectrometer (Jasco V-530, Japan Servo Co., Ltd., Tokyo, Japan).

### 2.5. Fatty Acids Determination 

The fatty acids profile of blackberry and raspberry leaves was determined using a gas chromatograph Perkin Elmer Clarus 500 (Waltham, MA, USA) according to the method described by [28]. The fatty acids present in the samples underwent the following two-step process: first, they were converted into methyl esters and then separated on a TRACE TR-Fame capillary chromatographic column featuring a highly polar stationary phase (Thermo Electron, Waltham, MA, USA), and dimensions of 60 m × 0.25 mm × 0.25 µm film. The detection of fatty acids was carried out using a flame ionization detector (FID), and their identification and quantification were achieved via a reference to standard chromatograms. 

### 2.6. Liposoluble Antioxidants

The extraction of liposoluble antioxidants from blackberry and raspberry leaves followed the procedure previously described [29]. Prior to extraction, a saponification step was necessary, involving the hydrolysis of samples with an ethanolic potassium hydroxide solution in a water bath for 30 min at 80 °C. The extraction was performed with petroleum ether.

Xanthophylls (lutein, zeaxanthin, astaxanthin, and cantaxanthin) were analyzed using an HPLC method and a liquid chromatograph (Perkin Elmer 200 series, Shelton, CT, USA) with a UV detector (detection at 450 nm). The chromatographic conditions involved a mobile phase of 10% water, 15% methanol and 75% acetone at a flow rate of 0.5 mL min^−1^ and a C18 reversed-phase column (5 µm, 250 × 4.60 mm i.d.) (Nucleodur, Macherey-Nagel, Germany).

The determination of vitamin E isomers was assessed using a liquid chromatograph (Vanquish Thermo-Electron Corporation, Waltham, MA, USA) and a PDA-UV detector at the wavelength 292 nm. The chromatographic conditions involved a mobile phase of 4% water and 96% methanol at a flow rate of 1.5 mL min^−1^ and a C18 reversed-phase column (5 µm, 250 × 4.60 mm i.d.) (Thermo-Electron Corporation, Waltham, MA, USA).

### 2.7. Watersoluble Antioxidants

The content of total polyphenols (TPCs) was determined using Folin–Ciocalteu’s spectrophotometric method as previously described [30]. The calibration curve of gallic acid was used to determine the total phenol content, and the results were reported as mg gallic acid equivalents per gram of the dried sample (mg GAE/g).

The determination of the total flavonoid content was performed using the aluminum chloride colorimetric method described by [31]. In a 10 mL volumetric flask, 1 mL of the methanolic extract of blackberry or raspberry leaves was mixed with 4 mL of aluminum chloride (AlCl_3_) and incubated at room temperature for 15 min. Subsequently, the absorbance of the resulting orange-yellow solution was measured at 410 nm against the blank using a UV-VIS spectrophotometer (Jasco V-530, Japan Servo Co., Ltd., Tokyo, Japan). The calibration curve was assessed with quercetin as a standard, and the flavonoid content was expressed as mg Quercetin equivalents (QE) per gram.

The profile of polyphenols was assessed using a liquid chromatographic method [32], a Vanquish Core HPLC system equipped with a DAD manufactured by Thermo Fisher Scientific (Bremen, Germany), and a BDS HyperSil C18 column (250 × 4 mm, 5 µm particle size) from Thermo Fisher Scientific (Bremen, Germany). The chromatographic method involves a binary gradient comprising acetic acid (1%) in distilled water (*v*/*v*) as solvent A, methanol as solvent B, and acetonitrile as solvent C, with a flow rate of 0.5 mL/min, and an elution program as follows: 0–15 min: 5% solvent B, 5% solvent C; 15–20 min: 4% solvent B, 15% solvent C; 20–25 min: 3% solvent B, 25% solvent C; 25–40 min: 2% solvent B, 38% solvent C; 40–50 min: 5% solvent B, 5% solvent C. The standards of individual polyphenols were purchased from Sigma-Aldrich (Steinheim, Germany) and used for the identification and quantification of polyphenolic compounds.

### 2.8. Antioxidant Activity Analysis

The antioxidant capacity of blackberry and raspberry extracts was assessed using four different spectrophotometric methods for the determination of DPPH, ABTS, antioxidant capacity, and iron chelating ability. For DPPH, the calibration curve was performed using 6-hydroxy-2,5,7,8 tetramethylchroman-2-carboxylic acid (Trolox) and a spectrophotometer (Jasco V-530, Japan Servo Co., Ltd., Japan) with the results expressed as mmol Trolox equivalents/kg of the sample. The scavenging potential of plant extracts against the ABTS radical was determined by measuring the absorbance at 734 nm (Jasco V-530, Japan Servo Co., Ltd., Japan) using ethanol as a blank. The evaluation of the total antioxidant capacity of the extract using the phosphomolybdenum method was assessed by measuring the absorbance of the samples at 695 nm, with the results expressed as ascorbic acid equivalents.

The chelation of ferrous ions by blackberry and raspberry leaf extracts was estimated. Briefly, 1 mL of the methanolic leaves extract (1:10, *w*/*v*) was mixed with 1.6 mL of deionized water in a 10 mL volumetric flask. A volume of 0.06 mL of 2 mM FeCl2 solution was added and allowed to stand at room temperature for 3 min when 0.12 mL of a 5 mM ferrozine solution was added. The mixture was vigorously shaken and then allowed to stand at room temperature for 10 min. Subsequently, the absorbance of the purple-colored complex formed was measured at 562 nm using a UV-VIS spectrophotometer (JASCO V 530, Japan Servo Co., Ltd., Japan) and comparing it to a blank sample. The results were expressed as mg disodium ethylenediamine tetra-acetic acid (EDTA-Na2) equivalents per g of the sample (equiv. mg EDTA/g).

The superoxide radical scavenging activity of leaves was based on the method described by [33]. The absorbance was measured at 560 nm with a spectrophotometer (Jasco V-530, Japan Servo Co., Ltd., Japan) against the blank samples. The increased superoxide anion scavenging activity was indicated by the decreased absorbance of the reaction mixture. The percentage inhibition of superoxide anion generation was calculated using the following formula:

% Inhibition = (AC − AS) × 100/AC,(1)

where AS is the absorbance of the sample, and AC is the absorbance of the control (ascorbic acid). 

The ability of the leaf extracts to annihilate the hydroxyl radical was evaluated according to the method described by [33]. The absorbance of the mixture was measured at 532 nm using ascorbic acid as a control. The results were expressed as the percent inhibition of the deoxyribose attack using the following formula:

% Inhibition = (AC − AS) × 100/AC,(2)

where AS is the absorbance of the sample, and AC is the absorbance of the control (ascorbic acid). 

### 2.9. Lipid Peroxidation Inhibition Assay 

In order to study the potential of the leaf extracts to inhibit lipid peroxidation in meat, a method for iron-induced lipid oxidation in chicken breasts was used. Breast samples were collected from broiler chickens fed with a conventional diet. A 2 mL aliquot of the breast samples’ homogenate prepared with a previously described method [33] was mixed with or without plant methanolic extracts (at a concentration of 1000 mg L^−1^) and in a total volume of 4 mL. Peroxidation was initiated by adding 0.2 mL of FeCl_2_ (100 µM) and 0.2 mL of ascorbic acid (500 µM) to the mixture. The mixture was incubated at 37 °C for 60 min.

The lipid oxidative status of breast meat was evaluated by measuring thiobarbituric acid reactive substances (TBARS) using a spectrophotometer (Jasco V-530, Japan Servo Co., Ltd., Japan) against a standard curve obtained with 1,1,3,3-tetramethoxypropane. The results were expressed as the µg MDA per kilogram sample (µg MDA/kg).

### 2.10. Static In Vitro Digestion of the Blackberry and Raspberry Leaves

The simulation of in vitro digestion processes was assessed following the static method protocol developed by INFOGEST [34]. Three saline solutions were prepared in order to simulate salivary (SSF), gastric (SGF), and intestinal (SIF) fluids. The protocol involved the three phases of sequential oral, gastric, and intestinal digestion, each requiring the addition of specific enzymes. Briefly, 5 g of the samples was mixed with 3.5 mL of simulated salivary fluids (SSFs) and 0.5 mL of α-amylase (prepared in SSF; final concentration 75 U/mL) previously heated at 37 °C, followed by 25 µL of a 0.3 M calcium chloride solution and 975 µL of distilled water. The mixture was incubated at 37 °C for 2 min. In order to simulate the gastric phase, 7.5 mL of the simulated gastric fluids (SGFs), which was pre-heated to 37 °C, and 1.6 mL of a pepsin solution (prepared in SGF; final concentration 2000 U/mL) were added to the oral bolus deriving from the simulated oral phase. Then, 5 µL of 0.3 M calcium chloride solution was further added. The pH was adjusted to 3 with 6 M HCl and distilled water added to obtain a final volume of 10 mL. The mixture was then incubated at 37 °C for 2 h. To simulate the intestinal phase, 11 mL of simulated intestinal fluids (SIFs), 5 mL of pancreatin 800 U/mL (prepared in SIF; final concentration 100 U/mL), 2.5 mL of bile salts 160 mM (final concentration 10 mM), and 40 µL of CaCl2 0.3 M were added to the gastric chyme. Subsequently, the pH was adjusted to 7 by NaOH at 1 M, and water was added to reach a 1:1 (*v*:*v*) ratio with gastric chyme. The mixture was then incubated at 37 °C for 2 h. At the end of each stage of the simulation in vitro digestion, the samples were centrifuged at 4500 rpm for 15 min at 4 °C using a laboratory refrigerated centrifuge (2-16KL, Sigma Laborzentrifugen GmbH, Germany). From the obtained supernatants, 1 mL was analyzed by HPLC to establish the bioaccessibility index of individual polyphenols.

The bioaccessibility index (BI) is defined as the ratio between the concentration of phenolic compounds released in the simulated digestion compared to the concentration of phenolic compounds in the non-digested plant and was calculated using the following equation [35]:

BI = (FC × 100)/IC,(3)

where FC is the concentration of compounds released during digestion, and IC is the concentration of compounds in the plant matrix before digestion.

### 2.11. Statistical Analysis 

All measurements were performed in triplicate. The data obtained were analyzed using one-way ANOVA, followed by Tukey’s test (*p* = 0.05) using the XLSTAT software (v.19.01, Addinsoft, Paris, France). A lack of statistically significant differences between the examined groups is indicated by similar letters. Pearson’s correlation coefficient analysis was performed in order to investigate the relationships between antioxidant compounds, the scavenging capacity of selected berry leaves on active oxygen species, and the inhibition of lipid peroxidation, using Prism-GraphPad software v. 9.1.2 (San Diego, CA, USA).

## 3. Results

### 3.1. Proximate Composition and Mineral Content

The data regarding the proximate composition of the selected berry leaves are shown in Table 1. There was no difference in the crude fat content in the leaves of the blackberry and raspberry. It was shown that blackberry leaves had a significantly higher (*p* < 0.05) fiber content in their dry matter, while raspberry leaves registered a higher content of the protein compared to blackberry leaves but not at a statistically significant level (*p* = 0.198).

An imbalance of essential minerals disturbs the normal functioning of the human organism, which can cause several pathological conditions. Considering the importance of trace elements, we investigated the content of some minerals. The concentrations of these elements in the samples are summarized in Table 1. Among the discussed plants, the highest (*p* < 0.05) content of Ca was found in blackberry leaves, which also contained the most (*p* < 0.05) Cu and Mn. Raspberry leaves registered the highest (*p* < 0.05) concentrations of Fe and Zn.

### 3.2. Fatty Acids Composition

The individual fatty acid content of blackberry and raspberry leaves is shown in Table 2 and Figure S3.

There were significant differences (*p* < 0.05) between the two berry leaves for the analyzed fatty acids. Blackberry leaves had the highest level (*p* < 0.001) of α linolenic acid (C18:3n3), whereas raspberry leaves registered a higher content of octadecatetraenoic acid (C18:4n3) and eicosapentaenoic acid (C20:5n3) compared to blackberry leaves. The content of MUFA in raspberry leaves was almost double that in the blackberry leaves. Among the saturated fatty acids, the palmitic acid content was three times greater than that of stearic acid for both types of the analyzed leaves.

### 3.3. Antioxidants Composition

The contents of liposoluble antioxidants are presented in Table 3 and Figure S2. The results show that blackberry leaves contain a significantly greater number of total tocopherols (*p* = 0.007) than raspberry leaves, among which α-tocopherol is the major compound (*p* < 0.0001). In raspberry leaves, the major compound was γ-tocopherol, which was significantly higher (*p* < 0.0001) than in blackberry leaves. Moreover, the levels of xantophylls were significantly higher (*p* < 0.0001) in blackberry leaves than in raspberry leaves, with zeaxanthin appearing three times higher in blackberry leaves compared to raspberry leaves. The total polyphenols and total flavonoids registered significantly higher concentrations (*p*< 0.05) in raspberry leaves compared to blackberry leaves.

### 3.4. Antioxidant Activity Analysis

The antioxidant activity of the blackberry and raspberry leaves (Table 4) was analyzed using four different assay methods (DPPH, ABTS, antioxidant capacity, and iron chelating ability). The results show that raspberry leaves had the highest antioxidant activity (*p* < 0.05) due to their iron chelating ability, DPPH, and antioxidant capacity, while, for the determination of ABTS, no significant differences were noted between the raspberry and blackberry leaves.

**Figure 1:**
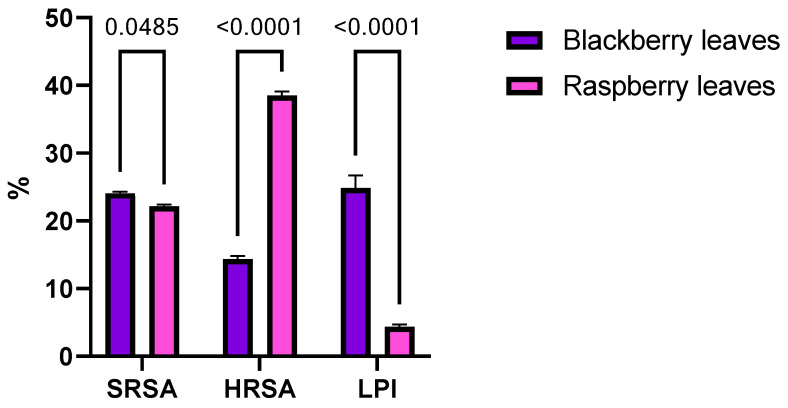
The scavenging capacity of selected berry leaves on active oxygen species (O_2_^•−^, ^•^OH) and the inhibition of lipid peroxidation. SRSA: superoxide radical scavenging activity, HRSA: hydroxyl radical scavenging activity, LPI: lipid peroxidation inhibition.

The free radical scavenging properties of blackberry and raspberry leaves were evaluated against hydroxyl (HO^•^) and superoxide radicals (O_2_^•−^), and the inhibition of lipid peroxidation was assessed afterward (Figure 1). 

Raspberry leaves had the highest (*p* < 0.0001) inhibition percentage for the free radical activity of ^•^OH, while blackberry leaves registered a higher (*p* = 0.0485) ability to inhibit O_2_^•−^free radical activity and a significantly higher (*p* < 0.0001) degree of inhibition of lipid peroxidation. 

**Figure 2:**
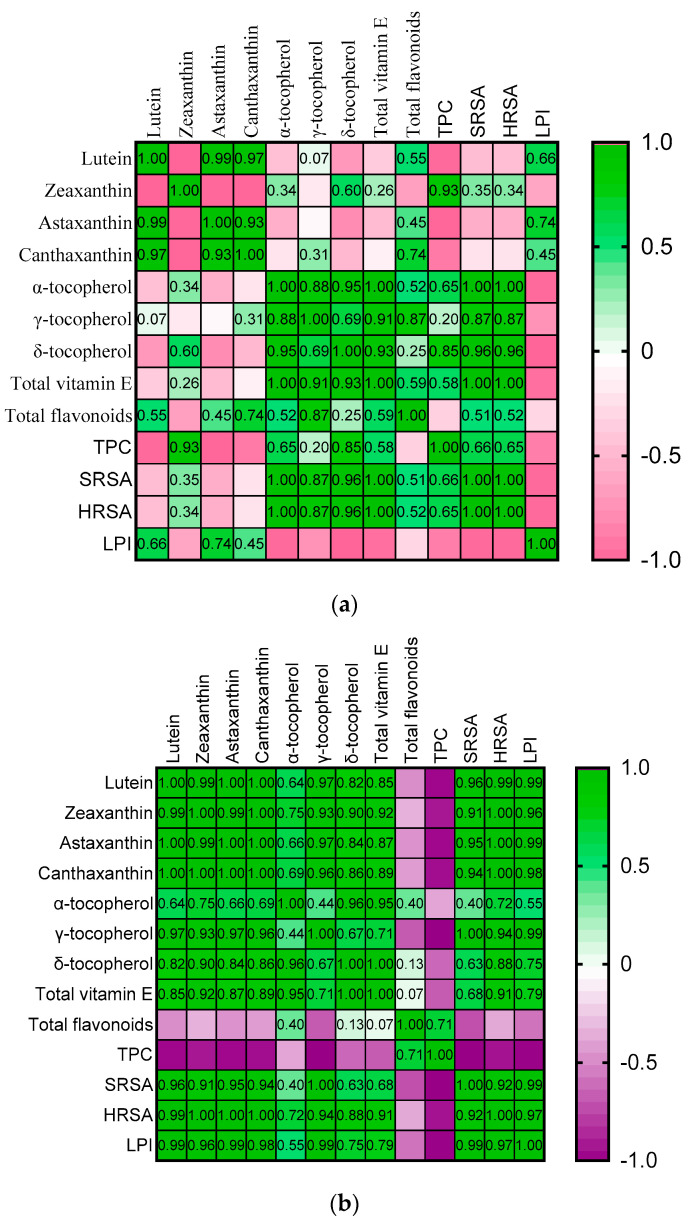
Pearson’s correlations between the antioxidant compounds, the scavenging capacity of selected berry leaves on active oxygen species (O_2_^•−^, ^•^OH), and the inhibition of lipid peroxidation. The positive correlations are highlighted in green and contain the correlation coefficient (r). The negative correlations are highlighted with pink (**a**) or purple (**b**). The darker the color is, the higher the correlation between the variables.

Correlations between the antioxidant content (lutein, zeaxanthin, astaxanthin, cantaxanthin, α-tocopherol, γ-tocopherol, δ-tocopherol, total vitamin E, TPC, total flavonoids) and the scavenging capacity of selected berry leaves on active oxygen species and the inhibition of lipid peroxidation were assessed as well (Figure 2a,b). In the case of raspberry leaves, the inhibition of lipid peroxidation was positively correlated with lutein, astaxanthin, and cantaxanthin. For blackberry leaves, a strong positive correlation with all the analyzed liposoluble antioxidant compounds was observed, while a strong but negative correlation was registered for the total polyphenol content and total flavonoid content. SRSA and HRSA were highly positively correlated with all the analyzed liposoluble antioxidant compounds in blackberry leaves, except for α-tocopherol, where a moderate correlation was obtained. In raspberry leaves, SRSA and HRSA were highly positively correlated with all the analyzed vitamin E isomers, while the total flavonoids and total polyphenols content were moderately correlated, and xantophylls were weakly and negatively correlated. 

### 3.5. In Vitro Digestibility

Eight aliquots of each type of leaves were recovered at different stages during GID (gastrointestinal digestion): 2 aliquots for undigested leaves; 2 aliquots after OP (oral phase, 2 min); 2 aliquots for GP (gastric phase, 120 min); and 2 aliquots for IP (intestinal phase, 120 min). In several published studies, the digestive procedure was performed in two phases (GP and IP), excluding the first digestive stage (OP). The oral phase holds particular significance in the digestion process as it involves the breakdown of the plant matrix containing phytocompounds during chewing. This phase exposes the components to salivary fluids and to the activity of α-amylase while also initiating an anaerobic atmosphere.

Certain similarities were observed in the polyphenol profile of these two types of leaves (Table 5 and Figure S1). Ferulic acid was the most abundant phenolic acid in both types of leaves, and its amount was also the highest in the intestinal phase. Among the flavonoids, epigallocatechin was the predominant one and followed the same behavior during the digestion of blackberry and raspberry leaves. Only gallic acid presented an increase throughout digestion in the blackberry leaves from 0.104 mg/g in the plant before digestion to 0.144 mg/g in the intestinal phase. In raspberry leaves, the amount of gallic acid in the intestinal phase was very close to the one in leaves before digestion, the intestinal bioaccessibility being 97.24%. Several phytochemicals that were observed on chromatograms from the lead extracts and in the intestinal phase of the digested leaves remained unidentified. The complex composition of the studied leaves was proven by the complex polyphenolic profile, with many other unidentified components with phenolic structures being present in the registered chromatograms (Figure S1). The reduced values for several bioaccessibility indexes can be explained by the degradation of phenolics or biotransformation processes during digestion. 

The findings from this study indicate that the concentration of phenolics increased following gastric digestion, thereby enhancing the bioaccessibility of the compounds.

## 4. Discussion

### 4.1. Proximate Composition and Mineral Content

The leaves of blackberry and raspberry plants are typically generated as byproducts during the cultivation of berries and are frequently discarded as waste in the process of growing the fruit. The utilization of agricultural and food-related waste materials, such as leaves, is not a novel strategy; however, it is gaining importance as an alternative means of obtaining raw materials with substantial economic potential [15,36]. Blackberry and raspberry leaves have been used in traditional medicine as herbal remedies throughout pregnancy [18,22]. Although environmental factors and harvest maturity can influence the content of leaves, it is essential to comprehend the chemical composition and antioxidant properties for the selective utilization of these species in the pharmaceutical or feed and food industries. In order to be used in the previously mentioned industries, several issues must be accounted for: the time of harvest (to protect the plant and fruit development), the maturity stage of plants for obtaining the best bioactive concentrations, and the preservation methods applied to minimize the loss of nutrients. Therefore, it is worthwhile to investigate the presence of bioactive compounds in these leaves and consider their potential applications.

The analysis of the proximate composition showed that the amount of crude protein in raspberry and blackberry leaves was higher compared with the concentrations reported by [37], which found 14.8% crude protein in raspberry leaves and 15.14% in blackberry leaves. The functional characteristics of food are linked to their fiber content [38], consisting of non-digestible carbohydrates that serve as a source of nutrition for symbiotic bacteria in the large intestine [39]. Dietary fiber plays a significant role in human health by contributing to the prevention and management of conditions such as diabetes, obesity, coronary heart disease, and colorectal cancers [40]. In our study, the levels of crude fiber found in raspberry and blackberry leaves were in accordance with the results noted by [37], who reported a fiber content of 18.28% for blackberry and 16.48% for raspberry plants.

Minerals are indispensable nutrients for organisms, including humans, to perform vital functions essential for life and well-being. Minerals have a significant function in the activation of enzymes that are responsible for cell metabolism and antioxidant mechanisms [41]. Important concentrations of minerals were found in the leaves of blackberry and raspberry plants. The results of this study are in agreement with the ones reported by [19], who reported an iron content between 117.7 mg/kg and 240.2 mg/kg in raspberry buds, which is higher compared to the same study in terms of the Cu, Mn, and Zn content in blackberry leaves. In this study, the content of Mn in the analyzed leaves was around ten times higher than the concentrations observed by [19] in the buds of blackberry and raspberry plants. According to the same authors, plant genotype, its organ, its physiological maturity, as well as climate and soil conditions may influence the variation in the mineral composition of the plant material. It is important to mention that the blackberry and raspberry from which the research material was collected were not fertilized as wild-growing plants. Therefore, the amount of ash in the dry matter of the blackberry and raspberry leaves could result from weather conditions.

### 4.2. Composition of Fatty Acids

The scientific data reported in the literature regarding the fatty acid content of blackberry and raspberry plants are limited and focused on the composition of the seed oil as a rich source of PUFA. Luo et al. [42] studied the fatty acid composition of several berry seed oils and observed that the linoleic acid content was the greatest among all unsaturated fatty acids and accounted for over 50% of the total fatty acids in the blackberry, red raspberry, and blackberry seed oils. In this study, similar results were obtained for blackberry leaves, whereas for raspberry leaves, the content of linoleic acid was close to the values reported by Bederska-Łojewska et al. [43]. Piasecka et al. [44] reported that linoleic acid and α-linolenic acid are the most common fatty acids in berry oils, classified as essential fatty acids. They must be obtained through dietary intake because the human body lacks endogenic production. The result of this study shows that the proportion of unsaturated fatty acids in raspberry leaves (66.53%) is higher than the result found by Chwil and Kostryco [45], where a content of 46.64% was reported. 

Blackberry leaves were found to have a higher content of PUFA compared to raspberry leaves, which was also noted in fruits of the selected plants in a study conducted by Zorzi et al. [46]. PUFAs are not synthesized within the human body because the enzymes necessary for forming double bonds in the fatty acid chain at a position beyond C-9 are absent [47]. In the human body, n-3 and n-6 fatty acids are integral components of cell membrane phospholipids, and their proportions in tissues largely rely on dietary intake. Essential fatty acids constitute a fundamental building block for cell structures and are recognized for their antiarrhythmic [48], anticoagulant [49], antiatherosclerotic, and anti-inflammatory properties [50], as well as their ability to enhance vascular endothelial function [51]. Plants rich in PUFAs show high nutritional value as they are recommended by FAO/WHO as an SFA replacement in the diet [52]. In order to assess the nutritional value of fat, the ratios of PUFA to SFA are commonly used [53]. In the present study, PUFA/SFA ratios were close to 2.6 in blackberry leaves and 1.4 in raspberry leaves. Dietary ratios of PUFA to SFA above 0.45 are recommended in the human diet [52] to prevent the development of cardiovascular disease and some other diseases, including cancer [54].

### 4.3. Antioxidants Composition

Extensive phytochemical studies have established the presence of a wide range of secondary metabolites within blackberry and raspberry leaves. These leaves are abundant in tannins and flavonoids, phenolic acids, triterpenes, mineral salts, and vitamin C, as reported in previous research [55]. The second most abundant category in raspberry leaves is flavonoids. The quantity of flavonoids in raspberry leaves is notably greater than in the fruit itself, where flavonoids represent only a small portion of the bioactive compounds [56]. Flavonoids possess antioxidant properties that serve to protect plants from a range of biotic and abiotic stresses. The secondary metabolic pathways in plants play a crucial role in responding to oxidative stress, leading to the production of flavonoids. Additionally, flavonoids in leafy plants play a significant role in acting as a protective screen against intense sunlight exposure [57]. According to Gudej [58], the flavonoid content in the raspberry leaf varies from 0.46% to 1.05% (*w*/*w*) and in blackberry leaves from 0.14% to 0.31%. In this study, similar values were obtained for the total flavonoids in raspberry leaves (1.06%), and higher values were noted in blackberry leaves (0.596%). Oszmianski et al. [59] showed that the flavonoid fraction emerges as the predominant phenolic group, comprising nearly 11% of the total weight of the extract powder of berry leaves. 

The total polyphenol content found in this study in raspberry leaves was 26.196 mg/100 g, which is much higher than the values reported by Ponder and Hallmann [60], which determined the polyphenols in different raspberry cultivars and observed a content between 0.88 and 1.51 mg/100 g. In the same study, it was shown that berry leaves, in addition to being a valuable antioxidant source, contained substantially higher levels of polyphenols compared to the fruit. Paczkowska-Walendowska et al. [61] also observed that the blackberry leaf extract has elevated concentrations of total polyphenols and increased antioxidant activity when compared to blackberry pomace. Furthermore, studies have explored whether the age of leaves might influence the total phenolic content, and it was observed that young leaves, typically derived from the upper portions of shoots or stems, exhibit considerably higher TPC levels compared to their older counterparts from the lower sections of shoots or stems [14]. Polyphenols not only exhibit antioxidant properties but can also modulate the intracellular redox balance through alternative mechanisms. These mechanisms include the inhibition of pro-oxidative enzymes like lipoxygenase [61]. 

The carotenoid analysis of raspberry and blackberry leaves showed that these leaves contain important amounts of pigments with antioxidant properties. The results of this study are higher than the ones reported by Ponder and Hallmann [60]. This could be caused by the different extraction procedures, which in this case was repeated four times with different solvents and was preceded by a saponification step. The quantity of carotenoids in raspberry and blackberry leaves may also be influenced by the chlorophyll content. Higher concentrations of chlorophyll in the leaves correspond to increased levels of carotenoids. Chlorophyll plays a role in the function of carotenoids, as these compounds are primarily produced by plants to protect the photosynthetic system from photooxidation. Carotenoids are synthesized through the general biosynthetic pathway within plant chloroplasts [60]. A similar observation was reported by Shen et al. [62], who investigated the influence of elevated UV-B radiation on carotenoid accumulation and its overall antioxidant capacity in tobacco (*Nicotiana tabacum* L.) leaves and observed a positive correlation between higher chlorophyll levels and an increased content of beta-carotene.

Tocopherols, the most potent fat-soluble antioxidants produced by higher plants, are typically found in various plant tissues, including seeds, fruits, leaves, and roots [63]. The available scientific literature provides limited information on the tocopherol content of blackberry and raspberry plants, only reporting data regarding the vitamin E composition of seed oil. A study conducted on several berry seed oils [44] found that γ-tocopherol is the main tocopherol present in blackberry and raspberry seed oil. In this study, a similar result was obtained for raspberry leaves, in which γ-tocopherol registered the highest concentration of all the analyzed isomers of vitamin E. In blackberry leaves, α-tocopherol was the dominant tocopherol present in the extract. Due to its specific chemical structure, tocopherol possesses unique physiological functions and bioactive potential, with particular emphasis on α-tocopherols, which represent the biologically active form of vitamin E. Vitamin E plays a powerful role in protecting polyunsaturated fatty acids (PUFAs), low-density lipoprotein (LDL), and other components of cell membranes against the oxidative damage caused by free radicals [64]. Tocopherols are known to protect against degenerative diseases, with a notable focus on Alzheimer’s and Parkinson’s diseases [65]. While the proper uptake of vitamin E is crucial from a nutritional perspective, α-tocopherol stands out as the most effective antioxidant in the human system, being the only tool capable of meeting the human body’s requirements for vitamin E [66].

### 4.4. Antioxidant Activity Analysis

The consideration of antioxidant activity is essential when evaluating the nutritional value of fruits and vegetables. Extensive research has been conducted and well-documented to highlight the substantial health benefits associated with diets rich in bioactive compounds [67,68]. The antioxidant properties of plants can be affected by a multitude of antioxidative mechanisms involving various chemical compounds within the plant material, along with their intricate interactions, which can include synergistic or antagonistic effects. As a result, it is necessary to employ a range of different methods, each with distinct mechanisms of action, in order to accurately assess antioxidant activity [69]. Using different in vitro screening assays to evaluate the antioxidant properties of blackberry and raspberry leaves gives better insight into their potential opportunities for health improvement and further applications. In this study, four in vitro screening antioxidant assays, DPPH, ABTS, antioxidant capacity, and iron chelating ability, were used with the aim of evaluating the antioxidant potential of leaves. 

The results of this study showed higher values of the antioxidant capacity when using DPPH than the ABTS method for blackberry and raspberry leaf extracts, which is in agreement with the observations of [19]. However, the findings from this study reveal that the DPPH levels in leaf extracts are higher than those observed in a previous study [19]. Specifically, the range recorded was 214.1–242.6 mmol/kg for raspberry leaves and 237.6 mmol/kg for blackberry leaves in the earlier study, whereas the current study indicates a greater level of DPPH in the extracts. In addition, ref. [14] reported that the leaves of blackberry and raspberry plants exhibited elevated antioxidant capacities and contained a greater total phenolic content in comparison to their fruit tissues. Moreover, they suggested that as the leaves aged, there was a decrease in both the total phenolic content and antioxidant potential. 

The variations observed in the antioxidant capacity behaviors of the studied samples could be attributed to differences in the mechanisms assessed by the employed techniques. For instance, in the ABTS method, the measurement focuses on the antioxidant capacity to donate electrons and reduce the ABTS^•^+ radical. Similarly, the DPPH protocol relies on the antioxidant capacity associated with transferring hydrogen atoms to radicals [70].

The extracts of blackberry and raspberry leaves, due to their significant concentration of antioxidants and other biologically active compounds, exhibit an inhibitory effect on the generation of reactive oxygen species (ROS). Our results are consistent with the results published previously by [71], who conducted a study on different berries cultivars and observed that blackberries had the highest antioxidant capacity against O_2_^•−^, ^•^OH and H_2_O_2_. Superoxide radicals, which are extremely reactive oxygen species, are released from cells in response to regular aerobic metabolism. Research has shown that superoxide radicals trigger the formation of more dangerous reactive oxygen species, such as hydrogen peroxide (H_2_O_2_) and hydroxyl radicals (^•^OH) [72]. The mutagenic potential of free radicals results from their direct interaction with DNA, and this interaction plays a significant role in the development of cancer [73]. Biochemical reactions can produce hydroxyl radicals. Specifically, superoxide radicals can be converted into hydrogen peroxide by superoxide dismutase, and in the presence of divalent metal ions like iron and copper, hydrogen peroxide can then generate highly reactive hydroxyl radicals. The hydroxyl radical, ^•^OH, is recognized for its extreme reactivity [74], with a short lifetime. It stands out as the most destructive among oxygen-based free radicals, targeting essential cellular components like proteins, nucleic acids, and membrane phospholipids. Studies have shown that berries contain anthocyanins, which are known to have phenolic hydroxyl groups attached to their ring structure, giving them antioxidant properties and allowing them to effectively neutralize reactive oxygen species (ROS) [75].

### 4.5. Lipid Peroxidation Inhibition Assay 

Previous studies have demonstrated that the consumption of berries may lead to a reduction in oxidative stress. This reduction can be attributed to the berries’ potential to influence the oxidation of proteins and lipids and enhance overall antioxidant levels [76]. In this study, blackberry leaves had a more pronounced effect in inhibiting lipid peroxidation in breast meat compared to raspberry leaves. Food manufacturers are using antioxidants to stabilize food lipids, preventing the quality degradation of products. In health-related areas, antioxidants play a crucial role in promoting well-being by protecting the body against oxidative damage. In a study conducted by [77], pork burger patties were enriched with blackberry extracts in order to test the oxidation of the muscle proteins (by measuring the protein carbonyl content) for 12 days (2 °C) after cooking. It was shown that supplementation with the blackberry extract significantly inhibited the formation of carbonyls during cooking. Other studies reported that berry pomace extracts have an inhibitory effect on the lipid oxidation of meat during refrigerated storage, and this may be attributed to their bioactive compounds that provide the main contribution to antioxidant activity [78,79]. Moreover, extracts of raspberry leaves were used to stabilize sunflower oil during accelerated storage, and [80] reported that the extracts of raspberry leaves not only represent a good source of natural antioxidants due to their high scavenging activity toward chemically generated superoxide radicals but also the high thermal stability of the extract shows an added advantage at high processing temperatures, contrary to synthetic antioxidants.

### 4.6. In Vitro Digestibility

Polyphenols found in foods are recognized as potential health promotors. However, these benefits rely on their bioaccessibility within the gastrointestinal tract. In order to confer health benefits, polyphenols must be released from the food matrix during digestion and become bioaccessible in the gastrointestinal tract. Subsequently, these compounds need to undergo a metabolization process to reach the target tissue, where they can exert their intended actions [81]. There is limited knowledge regarding the stability of these phenolic compounds during gastric and intestinal digestion, as well as their metabolism. It is also suggested that certain phenolic fractions are absorbed, while other fractions may traverse the digestive system and undergo microbial transformations upon reaching the colon [82]. Moreover, the intestinal microbiota possesses the ability to metabolize polyphenols with high molecular weight, transforming them into metabolites that exhibit enhanced biological activity [83].

In the assessment of the bioaccessibility and bioactivity of food molecules or natural products, in vitro digestion models play a crucial role in providing predictive insights. These static methods offer a straightforward and practical approach to studying various types of molecules with a broad applicability in nutritional, pharmaceutical, and toxicological fields. Furthermore, static models can be validated, mitigating the challenges associated with the difficult reproducibility of dynamic in vitro systems and the inter-individual variations encountered in in vivo experiments [84]. Bioaccessibility refers to the amount of a compound that is released within the gastrointestinal tract and available for absorption. The significance of the bioaccessibility of these bioactive compounds lies in their ability to effectively influence specific metabolic processes, contributing to health improvement [85].

From our knowledge, no previous studies have investigated the bioaccessibility and stability of polyphenol compounds present in blackberry and raspberry leaves. The findings from this study show that each compound exhibits a different bioaccessibility in the gastric phases. Under the influence of gastric and intestinal conditions, including variations in pH and exposure to enzymes, the solubility of these compounds can undergo alterations, thereby affecting their bioaccessibility. In the context of phenolic acids, modifications observed during in vitro digestion may be attributed to factors such as oxidation or degradation. As shown previously in blackberries [86], gallic acid is one of the most bioaccessible phenolics in both types of leaves. While various studies have demonstrated that the release of phenolics typically initiates in the oral or gastric phase [87,88], the outcomes of this investigation reveal that the most significant release of phenolic compounds from blackberry and raspberry leaves occurs either during or after the gastric phase. The results of this study are consistent with those reported by [89], who reported the highest release of health-related phenolic compounds from raspberry fruits and seeds during the intestinal phase. The nature of this plant is a significant factor affecting the bioavailability of polyphenols. Plant cell walls serve as a barrier to digestion, and their breaking through crushing or mastication leads to an association of phenolics with dietary fibers, and a modulation of polyphenols relative to bioaccessibilities [90]. Dietary fibers serve as the primary carriers for phenolic compounds, influencing their bioaccessibility because fiber-entrapped polyphenols are difficult to extract and exhibit limited solubility in gastrointestinal fluids. Proanthocyanidins and hydrolyzable tannins with high molecular weights constitute over 75% of all ingested polyphenols [91]. These compounds may tightly bind to dietary fibers restricting their accessibility.

For both types of leaves, hydroxybenzoic acids registered the highest bioaccessibility index in the intestinal phase. The remarkable changes in hydroxybenzoic acid derivatives, the subclass that became the most predominant after digestion, are possible due to the fact that hydroxybenzoic acids can be formed either as a degradation product of anthocyanins or as a metabolite [92]. Anthocyanins are unstable in an alkaline environment, and within the intestinal tract, these compounds undergo hydrolysis or degradation, resulting in the formation of phenolic acids and aldehydes. Structural variations at the B ring level of anthocyanins play a crucial role in the degradation of cyanidin, leading to the formation of protocatechuic acid [93]. Benzoic acid derivatives have exhibited protective effects on cardiovascular health, demonstrated anticancer and anti-obesity properties, and shown an ability to inhibit inflammatory responses in inflammatory bowel diseases [94].

Blackberry and raspberry plants contain significant concentrations of ellagitannins. In vitro digestion studies have suggested that ellagitannins are generally quite stable under the acidic conditions (pH 1.8–2.0) of the stomach. Enzymes in this environment are unable to decompose or hydrolyze them into ellagic acid units. As hydrolyzable tannins, their absorption initiates in the small intestine, primarily in the jejunum, following hydrolysis to ellagic acid [95]. In this study, the bioaccessibility of ellagic acid in blackberry leaves increased from 11.01% in the oral phase to 78.43% in the intestinal phase, while for raspberry leaves, a 6.46% bioaccessibility index was noted in the oral phase and increased to 35.46% in the intestinal phase. The presence of bile salts and pancreatin during the intestinal phase may facilitate the liberation of ellagic acid from the leaf’s matrix. This contribution is attributed to their role in breaking down complex ellagitannins and other derivatives of ellagic acid, ultimately leading to the formation of free ellagic acid. Similarly, the potential contribution of bile salts and pancreatin has previously been emphasized in the context of releasing and enhancing the bioaccessibility of carotenes derived from processed carrots [96]. Studies involving both human and animal model systems have demonstrated the apparent biological activities of ellagitannins and their metabolite, ellagic acid. These findings suggest their efficacy in combating chronic diseases such as cancer and cardiovascular disease [97]. 

The most abundant compound in both types of leaves was ferulic acid, which is a hydroxycinnamic acid. According to a previous study [98], ferulic acid is absorbed nearly entirely prior to reaching the colon. Ferulic acid is recognized for its ability to stabilize anthocyanins, inducing co-pigmentation within the matrix even at pH 3.0–4.0, specifically during the gastric phase [99]. Despite this stabilizing function, it is important to note that ferulic acid is also an intermediate product in the catabolism of flavonoids. This includes its involvement in the spontaneous cleavage of the B-ring of cyanidin aglycone and protocatechuic acid, as well as the spontaneous carboxylation of caffeic acid within the gastrointestinal tract [86].

While in vitro antioxidant assays may not precisely reflect in vivo conditions, they prove to be extremely valuable for the initial evaluation of the antioxidant potential of natural products. Understanding the breakdown of food constituents during digestion is crucial, as the potential effectiveness of plant metabolites for human health largely depends on the bioavailability of these molecules.

## 5. Conclusions

The results of this study show the comprehensive antioxidant activity of raspberry and blackberry leaves, based on a complex antioxidant profile, free radical scavenging, metal-chelating activity, antioxidant capacity, the inhibition of lipid peroxidation, and the bioaccessibility of individual polyphenols in simulated gastrointestinal digestion. 

In blackberry and raspberry leaves, polyphenols showed important values of bioaccessibility. Hydroxybenzoic acids were the most bioaccessible compounds during the digestion of the two types of berry leaves, which is the fact that can be associated with the catabolism of anthocyanins and an increase in free phenolic acids in the digestive matrix. Moreover, it was shown that the bioaccessibility of polyphenolic compounds increased with the progression of digestion.

It can be assumed that the use of raspberry and blackberry leaves offers health benefits while simultaneously addressing agri-food waste concerns. Raspberry and blackberry leaves can be considered unexploited sources of natural antioxidants with a positive effect on controlling the lipid oxidative processes in chicken breast meat and with high biological potency in scavenging free radicals under in vitro systems. Moreover, understanding the bioaccessibility and fate of polyphenol compounds during digestion is essential because the potential effectiveness of plant metabolites for human health largely depends on the bioavailability of these molecules.

## Figures and Tables

**Table 1:** Proximate composition and mineral content in blackberry and raspberry leaves.

Item	Blackberry Leaves	Raspberry Leaves	SEM	*p*-Value
Dry matter (g/100 g dw)	91.66 a	92.30 a	0.521	0.598
Crude protein (g/100 g dw)	18.37 a	19.54 a	0.426	0.198
Crude fat (g/100 g dw)	1.897 a	2.057 a	0.057	0.182
Crude fiber (g/100 g dw)	20.48 b	18.18 a	0.600	0.028
Ash (g/100 g dw)	6.203 b	5.137 a	0.265	0.015
*Minerals*				
Copper (mg/kg dw)	6.506 b	4.089 a	0.547	0.0001
Iron (mg/kg dry dw)	115.6 a	172.5 b	12.82	<0.0001
Manganese (mg/kg dry dw)	80.63 b	75.23 a	1.289	0.006
Zinc (mg/kg dry dw)	23.81 a	46.14 b	5.002	<0.0001
Calcium (g/100 g dw)	1.013 b	0.660 a	0.080	0.0002
Phosphorus (g/100 g dw)	0.290 a	0.280 a	0.010	0.656

a, b—Means in rows followed by the same letter are not significantly different at the 5% level of probability (*p* < 0.05).

**Table 2:** Fatty acid profile of blackberry and raspberry leaves (% of total FAs).

Fatty Acids	C:D	Blackberry Leaves	Raspberry Leaves	SEM	*p*-Value
Capric acid	C10:0	0.581 a	0.422 b	0.036	0.0002
Lauric acid	C12:0	1.517 a	1.382 b	0.030	0.0001
Myristic acid	C14:0	1.257 a	0.910 b	0.078	<0.0001
Pentadecanoic acid	C15:0	0.297 b	0.341 a	0.010	0.002
Pentadecenoic acid	C15:1	0.914 a	0.262 b	0.146	<0.0001
Palmitic acid	C16:0	15.73 b	21.52 a	1.295	<0.0001
Palmitoleic acid	C16:1	3.089 a	2.957 b	0.033	0.019
Stearic acid	C18:0	5.176 b	7.472 a	0.514	<0.0001
Oleic acid	C18:1	6.311 b	12.511 a	1.387	<0.0001
Linoleic acid	C18:2n6	13.39 a	12.15 b	0.277	<0.0001
α Linolenic acid	C18:3n3	48.40 a	29.52 b	4.221	<0.0001
Octadecatetraenoic acid	C18:4n3	2.405 b	3.746 a	0.302	<0.0001
Eicosapentaenoic acid	C20:5n3	nd	1.216	0.273	-
Lignoceric acid	C24:0	nd	1.303	0.292	-
Nervonic acid	C24:1n9	nd	4.154	0.929	-
Σ SFA		24.56 b	33.35 a	1.966	<0.0001
Σ MUFA		10.31 b	19.88 a	2.140	<0.0001
Σ PUFA		64.20 a	46.64 b	3.926	<0.0001
Σ n-3		74.51 a	66.53 b	3.649	<0.0001
Σ n-6		50.80 a	34.48 b	0.277	<0.0001
n-6/n-3		13.39 a	12.15 b	0.020	<0.0001
PUFA/SFA		0.264 b	0.353 a	0.272	<0.0001

C:D, carbon number: double bonds number; nd, not detectable; SFA, saturated fatty acids; UFA, total unsaturated fatty acids; MUFA, monounsaturated fatty acids; PUFA, polyunsaturated fatty acids. The relative concentration of each fatty acid is reported as the gram of fatty acids/100 g of total fatty acids. a, b—Means in rows followed by the same letter are not significantly different at the 5% level of probability (*p* < 0.05).

**Table 3:** Antioxidants analyzed in the selected berry leaves.

Item	Blackberry Leaves	Raspberry Leaves	SEM	*p*-Value
Liposoluble antioxidants				
Lutein, mg/kg	547.1 a	260.8 b	64.10	<0.0001
Zeaxanthin, mg/kg	3041 a	1040 b	450.1	<0.0001
Astaxanthin, mg/kg	38.52 a	7.441 b	6.997	<0.0001
Canthaxanthin, mg/kg	3.038 a	1.120 b	0.437	0.001
α-tocopherol, mg/kg	143.3 a	32.85 b	24.74	<0.0001
γ-tocopherol, mg/kg	29.30 b	85.66 a	12.63	<0.0001
δ-tocopherol, mg/kg	7.303 b	31.27 a	5.398	<0.0001
Total vitamin E, mg/kg	179.9 a	149.7 b	7.239	0.007
Water-soluble antioxidants				
TPC, mg/g GAE	14.57 a	26.19 b	2.324	0.003
Total flavonoids, mg/g	5.961 a	10.60 b	1.050	0.0002

TPC, total polyphenols content. a, b—Means in rows followed by the same letter are not significantly different at the 5% level of probability (*p* < 0.05).

**Table 4:** Antioxidant capacity of blackberry and raspberry leaves.

Item	Blackberry Leaves	Raspberry Leaves	SEM	*p*-Value
Iron chelating ability, equiv. mg EDTA/g	7.913 a	10.56 b	0.622	0.004
DPPH, mmol eq trolox/Kg	349.4 a	694.9 b	63.32	0.002
ABTS, mmol eq trolox/Kg	196.9 a	198.5 a	4.706	0.890
Antioxidant capacity, mmol eq ascorbic acid/Kg	118.1 a	157.3 b	9.217	0.003

a, b—Means in rows followed by the same letter are not significantly different at the 5% level of probability (*p* < 0.05).

**Table 5:** Polyphenol profile (mg/g) of blackberry and raspberry leaves subjected to simulated in vitro digestion via the INFOGEST protocol.

Specification	Blackberry Leaves							Raspberry Leaves						
	BD	OP	BI (%)	GP	BI (%)	IP	BI (%)	BD	OP	BI (%)	GP	BI (%)	IP	BI (%)
**Phenolic acids**														
*Hydroxybenzoic acids*														
Gallic acid	0.104	0.028	26.68	0.023	22.50	0.144	137.76	0.409	0.024	5.89	0.090	22.07	0.398	97.24
Vanillic acid	1.711	0.420	24.54	0.690	40.32	0.913	53.38	8.111	0.811	10.00	1.780	21.94	3.518	43.37
Syringic acid	0.148	0.031	21.03	0.042	28.74	0.084	56.65	0.346	0.017	4.86	0.061	17.58	0.127	36.85
3-hydroxybenzoic acid	0.452	0.114	25.27	0.197	43.60	0.242	53.59	nd	nd	nd	nd	nd	nd	nd
Ellagic acid	0.293	0.032	11.01	0.031	10.51	0.229	78.43	1.831	0.118	6.46	0.183	9.98	0.649	35.46
Protocatechuic acid	0.006	0.001	20.81	0.001	16.88	0.005	83.96	0.016	0.001	9.20	0.004	26.17	0.010	59.14
*Hydroxycinnamic acids*														
Chlorogenic acid	0.203	0.031	15.53	0.066	32.65	0.058	28.81	0.905	0.072	7.90	0.208	22.99	0.554	61.17
Caffeic acid	0.020	0.004	22.32	0.009	45.08	0.015	75.39	0.071	0.013	18.69	0.030	42.13	0.062	88.09
Methoxycinnamic acid	0.077	0.007	8.77	0.011	14.65	0.035	45.10	0.102	0.011	10.82	0.026	25.84	0.066	64.77
Ferulic acid	3.673	0.208	5.67	0.185	5.04	2.853	77.67	9.924	0.856	8.63	1.039	10.47	6.035	60.81
Coumaric acid	0.033	0.011	33.77	0.016	46.90	0.024	72.18	0.135	0.020	14.78	0.044	32.45	0.101	74.93
Cinnamic acid	0.009	0.002	26.79	0.002	19.04	0.007	75.76	0.008	0.001	10.08	0.001	14.33	0.006	77.11
**Flavonoids**														
*Flavanols*														
Epigallocatechin	0.495	0.151	30.62	0.185	37.35	0.265	53.51	1.981	0.168	8.48	0.416	21.01	0.678	34.21
Catechin	0.074	0.026	35.42	0.046	62.73	0.041	55.71	0.551	0.081	14.78	0.132	23.97	0.341	61.91
Epicatechin	0.212	0.043	20.19	0.047	22.01	0.161	75.91	0.997	0.110	10.99	0.234	23.45	0.553	55.52
*Flavonols*														
Rutin	0.027	0.009	32.00	0.013	47.01	0.023	83.26	0.286	0.017	5.85	0.060	21.12	0.141	49.43
Quercetin	0.012	0.003	21.43	0.004	37.83	0.006	47.93	0.005	0.001	13.79	0.002	32.24	0.003	49.04
**Stilbene**														
Resveratrol	0.027	0.012	45.07	0.013	48.31	0.021	78.52	0.016	0.001	6.40	0.006	39.66	0.007	43.98

BD = before digestion, OP = oral phase, GP = gastric phase, IP = intestinal phase, BI = bioaccessibility index, nd = not determined.

## Data Availability

The data are contained within the article and supplementary material.

## Supplementary Materials

The following supporting information can be downloaded at: https://www.mdpi.com/article/10.3390/antiox12122125/s1, Figure S1: Chromatograms of the phenolic compounds at 270 nm in raspberry leaves before digestion (a), in the intestinal phase of raspberry leaves (b), in blackberry leaves before digestion (c) and in the intestinal phase of blackberry leaves (d). Peaks of identification: 1—gallic acid, 2—epigallocatechin, 3—catechin, 4—chlorogenic acid, 5—vanillic acid, 6—caffeic acid, 7—syringic acid, 8—3-hydroxybenzoic acid, 9—epicatechin, 10—rutin, 11—coumaric acid, 12—ellagic acid, 13—methoxycinnamic acid, 14—ferulic acid, 15—protocatechuic acid, 16—resveratrol, 17—quercetin, 18—cinnamic acid. Figure S2. Chromatograms of tocopherols in raspberry (a) and blackberry leaves (b). Peaks of identification: 1—δ-tocopherol, 2—γ-tocopherol, 3—α-tocopherol. Figure S3. Chromatograms of fatty acids in raspberry (a) and blackberry leaves (b). Peaks of identification: 1—capric acid, 2—lauric acid, 3—myristic acid, 4—pentadecanoic acid, 5—pentadecenoic acid, 6—palmitic acid, 7—palmitoleic acid, 8—stearic acid, 9—oleic acid, 10—linoleic acid, 11—α linolenic acid, 12—octadecatetraenoic acid, 13—eicosapentaenoic acid, 14—lignoceric acid, 15—nervonic acid.
