# Supplementary material for: Exploring the Antioxidant Potential of Blackberry and Raspberry Leaves: Phytochemical Analysis, Scavenging Activity, and In Vitro Polyphenol Bioaccessibility
Source: Antioxidants (Basel). 2023 Dec 16;12(12):2125. doi: 10.3390/antiox12122125 (PMC10740815; doi:10.3390/antiox12122125)
Supplement: Supplementary file 1 [file antioxidants-12-02125-s001.zip › antioxidants-2764695-supplementary.pdf]

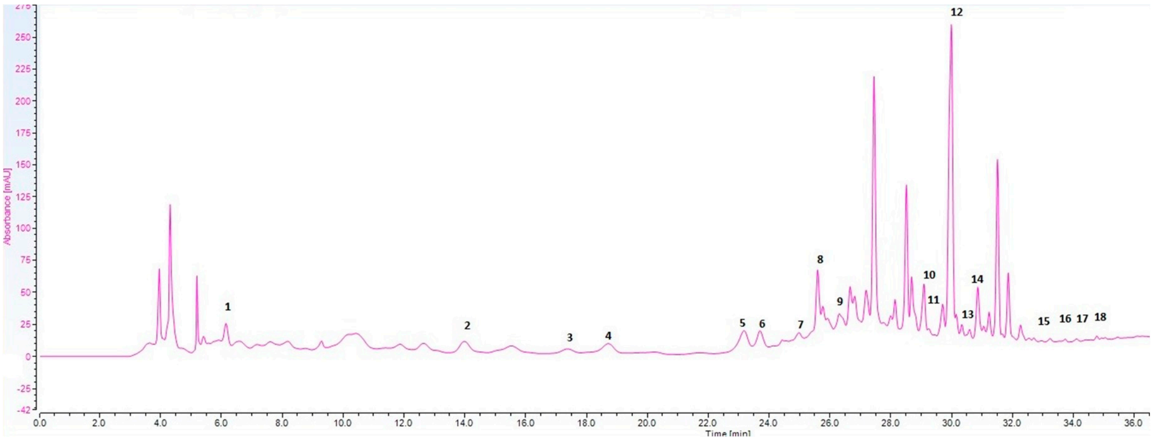
Absorbance [mAU]
Time [min]

(a)

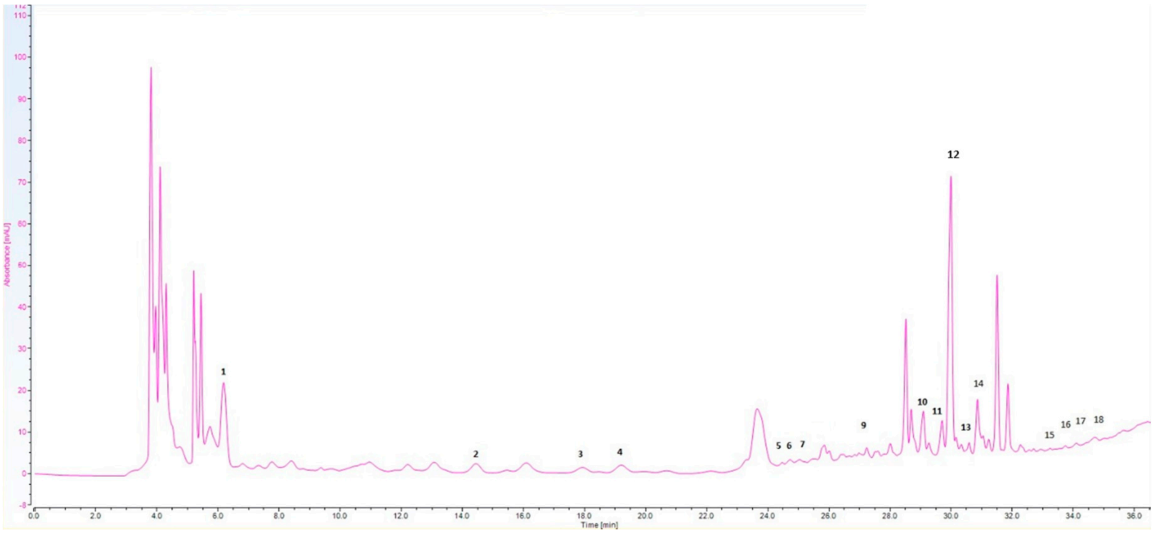
Absorbance [mAU]
Time [min]

(b)

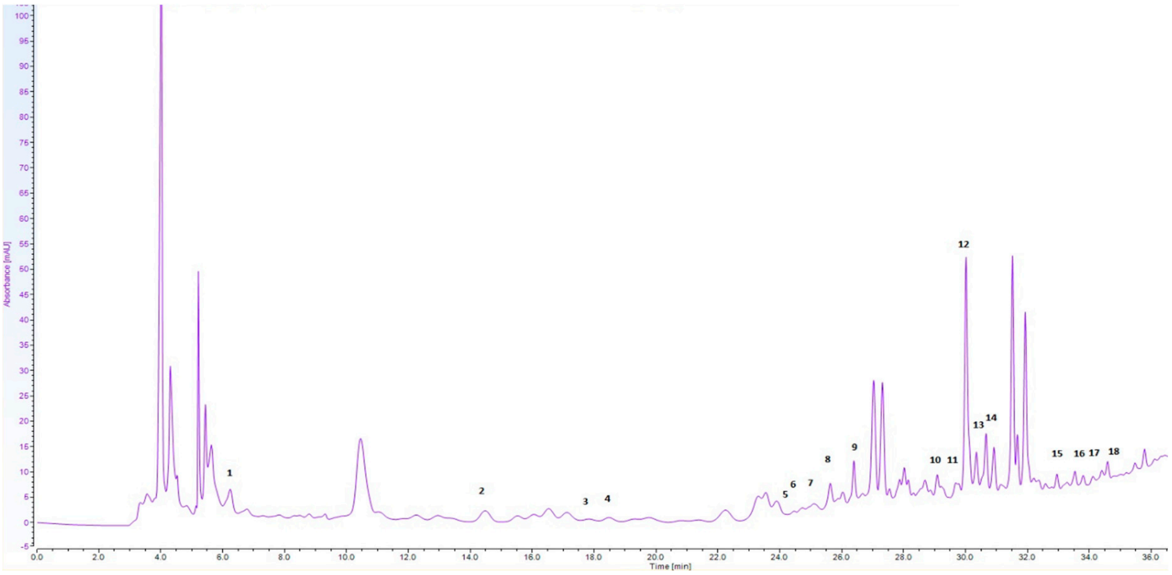
Absorbance [mAU]
Time [min]

(c)

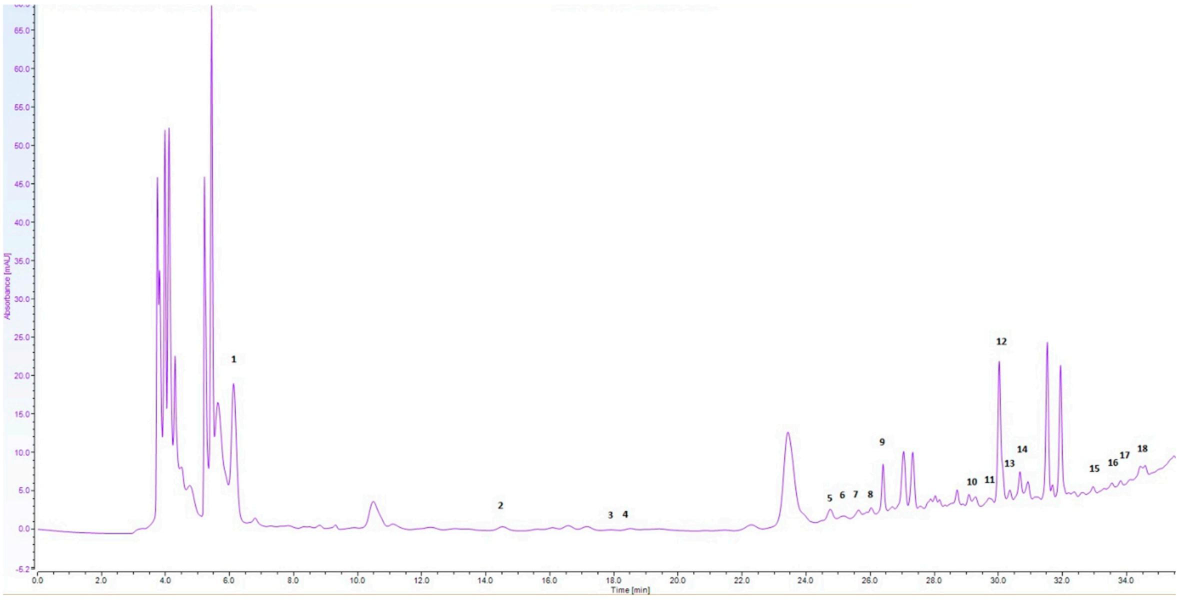

(d)

Figure S1. Chromatograms of the phenolic compounds at 270 nm in raspberry leaves before digestion (a), in the intestinal phase of raspberry leaves (b), in blackberry leaves before digestion (c) and in the intestinal phase of blackberry leaves (d). Peaks of identification: 1 - gallic acid, 2 - epigallocatechin, 3 - catechin, 4 - chlorogenic acid, 5 - vanillic acid, 6 - caffeic acid, 7 - syringic acid, 8 - 3-hydroxybenzoic acid, 9 - epicatechin, 10 - rutin, 11 - coumaric acid, 12 - ellagic acid, 13 - methoxycinnamic acid, 14 - ferulic acid, 15 - protocatechuic acid, 16 - resveratrol, 17 - quercetin, 18 - cinnamic acid.

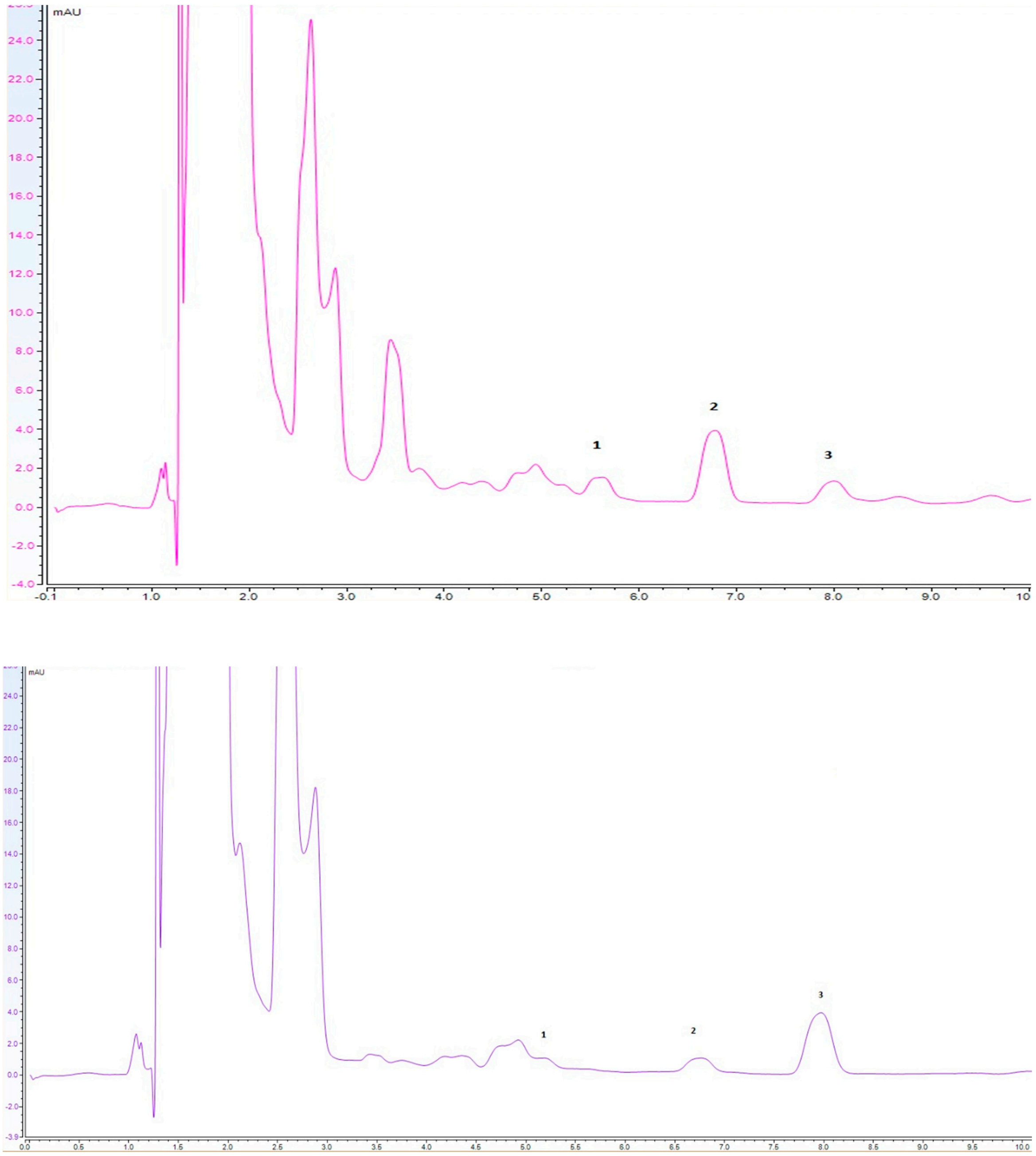

(a)

(b)

Figure S2. Chromatograms of tocopherols in raspberry (a) and blackberry leaves (b). Peaks of identification: 1 - δ-tocopherol, 2 - γ-tocopherol, 3 - α-tocopherol.

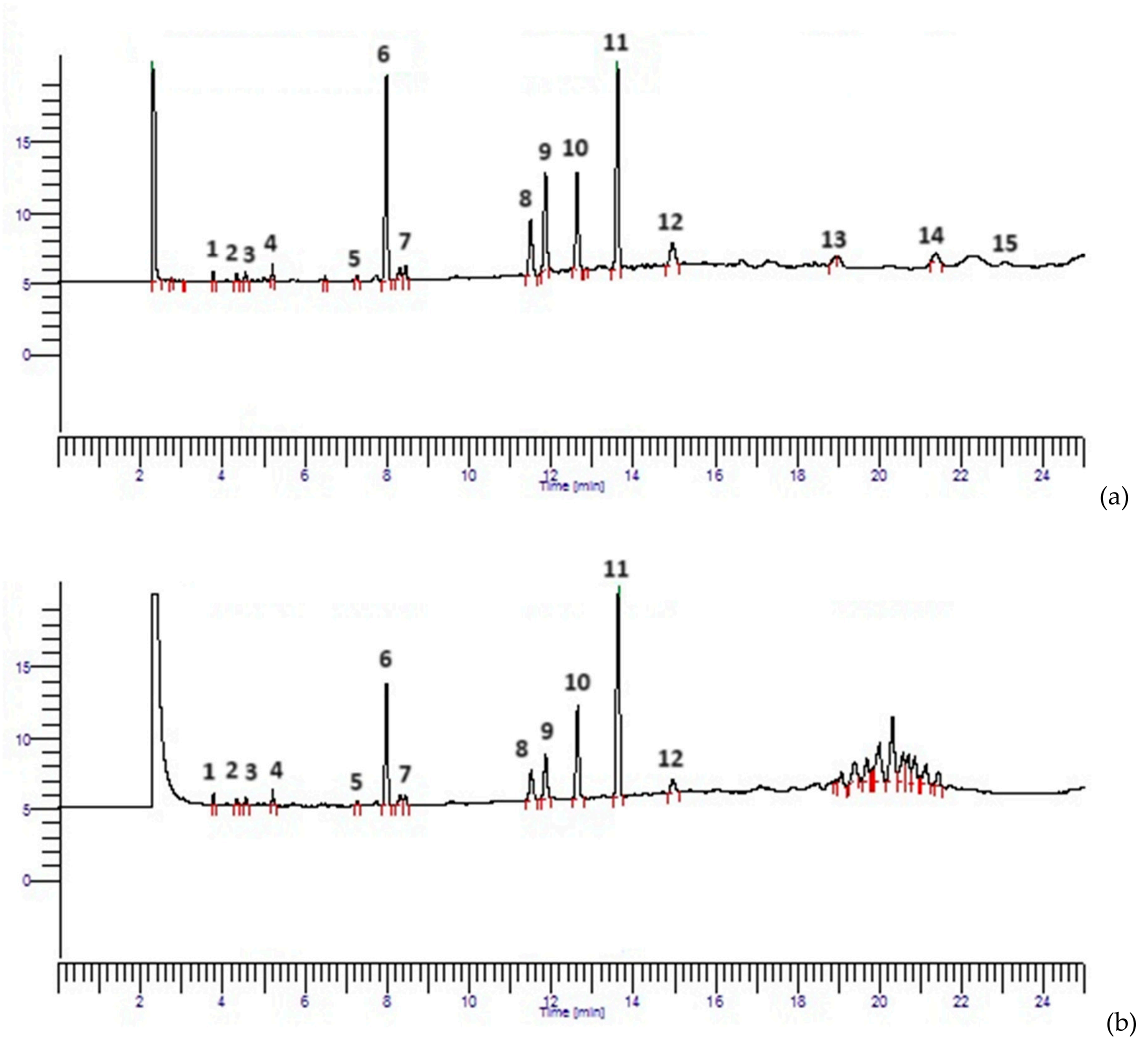

Figure S3. Chromatograms of fatty acids in raspberry (a) and blackberry leaves (b). Peaks of identification: 1 - capric acid, 2 - lauric acid, 3 - myristic acid, 4 - pentadecanoic acid, 5 - pentadecenoic acid, 6 - palmitic acid, 7 - palmitoleic acid, 8 - stearic acid, 9 - oleic acid, 10 - linoleic acid, 11 - α linolenic acid, 12 - octadecatetraenoic acid, 13 - eicosapentaenoic acid, 14 - lignoceric acid, 15 - nervonic acid.
